# Sensitivity-based virtual fields for the non-linear virtual fields method

**DOI:** 10.1007/s00466-017-1411-6

**Published:** 2017-04-28

**Authors:** Aleksander Marek, Frances M. Davis, Fabrice Pierron

**Affiliations:** Faculty of Engineering and the Environment, University of Southampton, Highfield, SO171BJ UK

**Keywords:** Virtual fields method, Sensitivity-based virtual fields, Inverse identification, Full field measurement, Elasto-plastic, Digital image correlation

## Abstract

The virtual fields method is an approach to inversely identify material parameters using full-field deformation data. In this manuscript, a new set of automatically-defined virtual fields for non-linear constitutive models has been proposed. These new sensitivity-based virtual fields reduce the influence of noise on the parameter identification. The sensitivity-based virtual fields were applied to a numerical example involving small strain plasticity; however, the general formulation derived for these virtual fields is applicable to any non-linear constitutive model. To quantify the improvement offered by these new virtual fields, they were compared with stiffness-based and manually defined virtual fields. The proposed sensitivity-based virtual fields were consistently able to identify plastic model parameters and outperform the stiffness-based and manually defined virtual fields when the data was corrupted by noise.

**Electronic supplementary material:**

The online version of this article (doi:10.1007/s00466-017-1411-6) contains supplementary material, which is available to authorized users.

## Introduction

Owing to the rapid diffusion of full-field deformation measurement techniques like Digital Image Correlation [26], there has been growing effort from the mechanics of materials scientific community to develop new testing methods based on more complex tests to improve the efficiency of constitutive model identification. This is particularly important for models including larger numbers of parameters like anisotropic elasticity and plasticity as well as heterogeneous materials. It is beyond the scope of the present article to review this topic in detail and the reader is referred to the following references for a more complete picture of this topic [2, 20, 22]. The two main techniques employed in the literature to extract the constitutive parameters from deformation maps are Finite Element Model Updating (FEMU) and the Virtual Fields Method (VFM). FEMU relies on the intuitive idea that parameters can be identified by updating the material parameters in a finite element model until the simulation matches the experiment [2]. An alternative is the Virtual Fields Method [20] which directly calculates the stresses from the measured strains, without a need to conduct forward calculations using FEM. Stress equilibrium is then evaluated in the global sense by means of the principle of virtual work and parameters adjusted until this equilibrium is respected.

The main advantage of the VFM over FEMU is its computational efficiency, particularly for non-linear problems. Recently, authors reported that the VFM was 125 times faster than FEMU for their particular application [30], so there is a definite motivation to choose the VFM over FEMU. However, the choice of the virtual fields (see Sect. 2) plays a crucial role in the VFM. In linear elasticity, manually defined virtual fields were used until the mid-2000s with mixed success [18]. In 2004, a systematic procedure for defining noise minimizing virtual fields was released which enabled the virtual fields to be automatically defined [1]. These automated virtual fields are now routinely used to solve linear elasticity problems, even for heterogeneous materials [11]. This success led them to be implemented on a commercial software platform, MatchID (v. 2.1, www.matchidmbc.be). However, for non-linear constitutive models, no automated approach for defining the virtual fields exists and this constitutes a bottleneck for the establishment of the VFM as the gold standard to inversely identify material properties from full field measurements [22].

In absence of an automated procedure to define virtual fields for non-linear problems, the VFM has been implemented with manually defined virtual fields. Elasto-plasticity was the first type of non-linear constitutive model to be tackled [8]. The non-linear virtual fields method has since been used to study a wide range of materials such as arteries [4], rubbers [10, 28, 29], composites [7], and metals [12–14, 17, 25]. As a result, many different non-linear constitutive model types were considered including hyperelasticity, elasto-plasticity, visco-elasticity, and anisotropic plasticity. While manually defining the virtual fields for non-linear problems has been successful, there are several drawbacks. The selection of manual virtual fields relies on the expertise of the investigator. In addition, the fields are generally static and do not evolve with the deformation. The only attempt so far at defining an automated procedure to define the virtual fields adapted the procedure for linear elasticity [1] to elasto-plasticity [19]. The virtual fields obtained were named *stiffness-based* virtual fields since they depend on the elasto-plastic stiffness matrix. However, the problem of systematically defining efficient and robust virtual fields for a general class of non-linear problems hinders the widespread diffusion of the VFM as a standard tool.

In this manuscript, a new type of automated virtual fields for non-linear problems is proposed. In Sect. 2, the virtual fields method is described in detail. Next, the automated virtual fields, the so-called *sensitivity-based* virtual fields, are derived introducing two variants based on total and incremental sensitivity maps. The performances of the proposed sensitivity-based virtual fields are then compared to stiffness based and manually defined virtual fields in Sect. 5 for the simulated experiment described in Sect. 4.

## Virtual Fields Method

For a solid that is subjected to quasi-static loading the principle of virtual work can be expressed as

1 $$\begin{aligned} -\int _V \varvec{\sigma }\left( \mathbf{x}, t \right) : \varvec{\varepsilon }^*\left( \mathbf{x}, t \right) \, dV + \int _{\partial V} \varvec{T} \cdot \varvec{u}^*\left( \mathbf{x}, t \right) \, dS = 0 \nonumber \\ \end{aligned}$$

where *V* is the volume of the solid, $\varvec{\sigma }$ is the stress tensor, $\varvec{u}^*$ and $\varvec{\varepsilon }^*$ are the virtual displacement and strain, respectively, and $\varvec{T}$ is the traction vector. Note that the stress tensor, virtual displacement, and virtual strain can vary in space and time. The stress field is calculated directly from the measured displacements (strains) using assumed a certain constitutive relation. The calculations are performed with a numerical implementation of the constitutive model, such as the radial-return algorithm for plasticity. Therefore, no forward problem solving using FEM is required. In Eq. 1, the virtual displacement does not have any physical meaning, and can be any function that is continuous and differentiable over the body. Likewise, the virtual strains do not have any link with the real strains but only serve as a spatial weighting functions (also sometimes called ‘test functions’). The virtual strain is calculated from the virtual displacement using the traditional strain-displacement relationship, $\varvec{\varepsilon }^* =1/2 \left( \nabla \varvec{u}^* +\nabla \varvec{u}^{* \mathrm {T}} \right)$. The first integral in Eq. 1 is the contribution of *internal virtual work* due to deformation and the second integral is the contribution from *external virtual work* due to externally applied loads.

Since in general, full-field measurements are only performed on the surface of the specimen some assumption on the material behaviour through the thickness must be made. In this manuscript, the assumption of plane stress is used since the samples are loaded in plane and are considered thin. However, alternative assumptions on the behaviour through the thickness are possible. Equation 1 can be re-written for a thin specimen in a state of plane stress:

2 $$\begin{aligned}&-h \int _S \varvec{\sigma }\left( x, y, t \right) \cdot \varvec{\varepsilon }^*\left( x, y, t \right) dS\nonumber \\&\quad +\,h \int _{\partial S} \varvec{T} \cdot \varvec{u}^*\left( x, y, t \right) dL = 0 \end{aligned}$$

where *h* is the thickness of the solid, *S* is the surface of the solid, and $\partial S$ is the boundary of the solid. In Eq. 2, the stress and virtual strain tensors have been written as vectors: $\varvec{\sigma }\left( x, y, t \right) = [\sigma _{11}, \sigma _{22}, \sigma _{12}]$ and $\varvec{\varepsilon }^{*} \left( x, y, t \right) = [\varepsilon _{11}^{*}, \varepsilon _{22}^{*}, 2\varepsilon ^{*}_{12}]$.

Full-field measurements such as digital image correlation [26] or the grid method [9] determine the displacement at a large number of discrete locations called measurement points. When the number of measurement points is large, the first integral in Eq. 2 can be well-approximated as a discrete sum using the mid-point rule. Often the distribution of the traction is unknown and only the resultant force, $\varvec{F}= \int \varvec{T} dA$, is measured using a load cell. When the resultant force is known, the external virtual work, $W^*_{ ext}$, can be directly calculated provided that the virtual displacements are constant over the area where the unknown traction distribution acts. As a result Eq. 2 can be re-written for any time *t* as

3 $$\begin{aligned} \left( \sum _{j=1}^{n{Pts}}\left( \varvec{\sigma }^{j} \cdot {\varvec{\varepsilon }^{*j}}\right) S^j \right) - W^*_{ ext} = 0 \end{aligned}$$

where *nPts* is the number of measurement points and $S^j$ is the area of the $j{\mathrm {th}}$ measurement point.

A constitutive relation must be used to calculate the stress from the strain data recorded during an experiment. The constitutive relation relates the measured strains to the stresses, $\varvec{\sigma } = \varvec{\sigma }\left( \varvec{\varepsilon }, \varvec{X} \right)$, where $\varvec{X}$ are the unknown constitutive parameters. In the case of linear elastic materials, the constitutive parameters can be identified by solving a set of linear equations. This occurs because the stress is a linear function of the strain. However, for non-linear constitutive relationships, the VFM no longer yields a set of linear equations. As a result, the identification process is based on the minimisation of a cost function with respect to the constitutive parameters. Using a suitable set of virtual displacements, the cost function can be defined as the least squares difference between the internal and external virtual work through time:

4 $$\begin{aligned} \varPhi (\varvec{X}, \varvec{\varepsilon })= & {} \sum _{i=1}^{nVF} \left[ \sum _{t=1}^{n{Time}} \right. \nonumber \\&\left. \left( \sum _{j=1}^{n{Pts}}\left( {\varvec{\sigma }^{j}\left( \varvec{\varepsilon }, \varvec{X} \right) \cdot \varvec{\varepsilon }^{*j}}^{(i)}\right) S^j- W^*_{ext}\right) ^{2} \right] \end{aligned}$$

where *nVF* and *n*Time are the numbers of independent virtual fields and time steps, respectively. The difference between the internal and external virtual work will be minimized, indicating that equilibrium has been satisfied, when the parameters have been correctly identified. Therefore, to identify the material parameters for a non-linear constitutive model, the process is iterative.

To identify the constitutive parameters, $\varvec{X}$, a set of virtual displacements and strains must be defined. There is an infinite number of virtual displacement fields that satisfy the principle of virtual work (Eqs. 1–3). In the case of a linear elastic model, when the number of independent virtual fields equals the number of unknowns a linear system is produced that when inverted gives the model parameters. For these linear systems, Avril et al. [1] have proposed a set of optimised virtual fields that minimise the influence of noise on the parameter identification. For non-linear problems, the situation is more complex. The number of virtual fields required for a successful identification is not necessarily equal to the number of parameters. However, the selected virtual fields need to activate the different parameters of the model, and provide a solution which is as robust as possible with respect to measurement noise. Until now, and except the effort reported in [19], the virtual fields were defined intuitively by the user. In this article, a new procedure is devised to automatically generate virtual fields to extract non-linear parameters with a view to increase the robustness from the procedure defined in [19].

## Theoretical development

As constitutive models become more complex, the number of model parameters tends to increase. The difficulty in identifying these parameters lies in determining where sufficient information on each parameter is coded in space (*x*, *y*) and time (*t*). The idea behind the proposed sensitivity-based virtual fields is that they will focus on regions that carry the most information about the constitutive parameters and follow them through time. By perturbing each model parameter, it is possible to determine the sensitivity of the stress to each parameter in the constitutive model. In a region where the stress change is significant, relevant information is encoded for identifying that parameter. The sensitivity of the stress field is chosen here as this particular field carries information in the VFM. Moreover, the stress field is the only quantity that depends directly on the constitutive parameters in the VFM, so using these stress sensitivity maps seems like a very natural idea to select areas with strong dependence to a given parameter. Since virtual strains can be seen as spatial weight functions, the resulting stress sensitivity map can be used to focus the identification on these critical regions where each model parameter has significant influence. Each model parameter will produce a different stress sensitivity map and therefore requires its own virtual field. Stress sensitivity based virtual fields following the idea outlined above are now derived. It is anticipated that by using these virtual fields to focus on regions where the signal is the most significant for a given parameter, identifiability of all parameters will be improved and the influence of noise on the identification will be reduced.

### Formulation of sensitivity-based virtual fields

To investigate the spatial sensitivity of the stress field to each model parameter, the stress sensitivity defined as

5 $$\begin{aligned} {\delta \varvec{\sigma }}^{(i)}\left( \varvec{\varepsilon }, \varvec{X}, t \right) = \varvec{\sigma }\left( \varvec{\varepsilon }, \varvec{X}, t \right) - \varvec{\sigma }\left( \varvec{\varepsilon }, \varvec{X}+\delta X_i, t \right) \end{aligned}$$

was calculated. In Eq. 5, $\varvec{X}$ is the vector of the model parameters, *i* denotes the $i\mathrm {th}$ model parameter in vector $\varvec{X}$, and *t* is the time step. By applying a small variation to a single model parameter, $\delta X_i$, it is possible to map in the stress field the most significant spatial changes associated with this model parameter. In regions where ${\delta \varvec{\sigma }}^{(i)}$ is close to zero, the varied parameter has a minimal effect on the stress, and conversely, large values of ${\delta \varvec{\sigma }}^{(i)}$ indicate that small changes in $X_i$ produce large changes in the stress.

To minimize the history dependence of the stress sensitivity, the incremental stress sensitivity for each model parameter, ${\delta \widetilde{\varvec{\sigma }}}^{(i)}$, is calculated as:

6 $$\begin{aligned} {\delta \widetilde{\varvec{\sigma }}}^{(i)}\left( \varvec{\varepsilon }, \varvec{X}, t \right) = {\delta \varvec{\sigma }}^{(i)}\left( \varvec{\varepsilon }, \varvec{X}, t \right) - {\delta \varvec{\sigma }}^{(i)}\left( \varvec{\varepsilon }, \varvec{X}, t-1 \right) \end{aligned}$$

By subtracting the stress sensitivity between two consecutive time steps, only the regions in the stress map that have changed during that particular time increment are highlighted. This is equivalent to a temporal finite difference of the stress sensitivity maps. Note that an incremental stress sensitivity must be calculated for every time step and constitutive parameter.

The idea is to use these incremental stress sensitivity maps as virtual strain maps. However, virtual displacements need to be defined to calculate the virtual work of external forces. Moreover, the virtual displacements are also chosen to eliminate certain unknown contributions of the traction forces at the boundary (this will be referred to as ‘virtual boundary conditions’ in the rest of the article). The objective is to define a set of virtual displacements such that their associated virtual strain maps ‘look like’ the incremental sensitivity stress maps. To accomplish this, a least-square projection approach, described next, was implemented.

To define the virtual displacement, $\varvec{u}^*$, from the incremental stress sensitivity defined in Eq. 6, a virtual mesh was implemented as it provides more flexibility to include virtual boundary conditions and ensures numerical stability compared to functions continuously defined over the whole field of view [20]. The domain, *S*, is broken into several virtual elements, collectively called a virtual mesh. Additional details on the virtual mesh can be found in “Appendix 1”.

The virtual strains at each measurement point are related to the virtual displacement at the four nodes of the virtual element that contains the point. A set of three linear equations can be written for each measurement point which relates the local virtual strains to the virtual displacements. When the equations for every element in the mesh are collected the following system of equations is produced:

7 $$\begin{aligned} {\delta \widetilde{\varvec{\sigma }}}^{(i)} = \varvec{B}\, {\varvec{u}^*}^{(i)} \end{aligned}$$

where $\varvec{B}$ is the global strain-displacement matrix which maps the virtual displacement at every node into virtual strains. There are $3 \times nPts$ equations with $2 \times nNodes$ unknowns for each model parameter. The virtual boundary conditions place constraints on the virtual displacement, ${\varvec{u}^*}^{(i)}$. When the displacement at a boundary is prescribed, the traction at the surface is generally unknown. To eliminate the contribution of this unknown traction to Eq. 3, the virtual displacement at these boundaries are set to zero. Often, the distribution of the traction is unknown and only the resultant force, $\varvec{F}= \int \varvec{T} dA$, is measured. In this case, a constant virtual displacement is applied on the boundary.

Enforcing the constraints on ${\varvec{u}^*}^{(i)}$, a modified global strain-displacement matrix, $\bar{\varvec{B}}$, is found. The virtual displacements are obtained by multiplying the pseudo-inverse of the modified global strain-displacement matrix with the incremental stress sensitivity,

${\varvec{u}^*}^{(i)} = \mathrm {pinv} \left( \bar{\varvec{B}}\right) {\delta \widetilde{\varvec{\sigma }}}^{(i)}$. The virtual strains, ${\varvec{\varepsilon }^*}^{(i)}$, also called the sensitivity-based virtual fields, are then calculated using the right hand side of Eq. 7. As a result, the virtual displacements are calculated so that the resulting virtual strains match the incremental stress sensitivity in a least-squares sense. Although ${\varvec{u}^*}^{(i)}$ and ${\varvec{\varepsilon }^*}^{(i)}$ must be calculated several times to perform the parameter identification, $\varvec{B}$ and $\mathrm {pinv} \left( \bar{\varvec{B}}\right)$ are only computed once because the virtual mesh and boundary conditions remain unchanged.

### Inverse parameter identification procedure

To identify the model parameters using the sensitivity-based virtual strains and displacements, the following cost function, $\varPhi$, is minimized:

8 $$\begin{aligned} & \varPhi (\varvec{X}, \varvec{\varepsilon }) = \sum _{i=1}^{n{Params}} \left[ \frac{1}{{\left( \alpha ^{(i)}\right) }^2} \right. \nonumber \\&\quad \times \left. \sum _{t=1}^{n{Time}}\left( \sum _{j=1}^{n{Pts}}\left( {\varvec{\sigma }^{j}\left( \varvec{\varepsilon }, \varvec{X}, t \right) \cdot \varvec{\varepsilon }^{*j}}^{(i)}(t)\right) S^j{-} W^*_{ext}(t)\right) ^{2}\!\right] .\nonumber \\ \end{aligned}$$

In Eq. 8, since the contribution of each parameter can vary greatly in magnitude, it is scaled by $\alpha ^{(i)}$. The scale factor, $\alpha ^{(i)}$, is calculated for each model parameter from the mean of the *n* highest internal virtual work (IVW) values where the IVW is defined as:

9 $$\begin{aligned} \mathrm {IVW}^{(i)}(t) =\sum _{j=1}^{n{Pts}}\left( {\varvec{\sigma }^{j}\left( \varvec{\varepsilon }, \varvec{X}, t \right) \cdot \varvec{\varepsilon }^{*j}}^{(i)}(t)\right) S^j \,. \end{aligned}$$

Scaling is necessary because the constitutive parameters are active over different time scales. As an example when attempting to identify the parameters in a plastic model, the parameters which capture the yielding behaviour will only be active for a short time period while hardening parameters will, in general, be active for much longer times. The suitability of this scaling method is validated in Sect. 5.

### Alternative virtual fields

To quantify the improvement in parameter identification provided by the sensitivity-based virtual fields, their results are compared with that from both uniform and stiffness-based virtual fields. The manually defined uniform virtual field applies a linear virtual extension in the $x_{2}$-direction and the resulting strain field is uniform. Since the virtual strain is uniform, the stress in the y-direction is integrated and compared directly with the resultant force (Eq. 2). The virtual displacements and strains which define this uniform virtual field are:

10 $$\begin{aligned} {\left\{ \begin{array}{ll} u_1^* = 0 \\ u_2^* = x_2 \end{array}\right. } \quad {\left\{ \begin{array}{ll} \varepsilon _{11}^* =0 \\ \varepsilon _{22}^* =1 \\ \varepsilon _{12}^* =0 \end{array}\right. } \end{aligned}$$

The most common approach for the non-linear VFM has been to manually define the virtual fields and therefore it is interesting to evaluate it against the sensitivity-based virtual fields.

Stiffness-based optimised virtual fields [19] were also implemented; these virtual fields are an earlier attempt at developing noise-optimised virtual fields for elasto-plasticity. The stiffness-based virtual fields were derived using the same noise minimization approach developed for virtual fields optimization in linear elasticity [1]. The stiffness-based virtual fields depend on the tangent stiffness matrix, $D^{ep}$, and scale each element’s contribution by its effective von Mises stress. The reason for scaling by the effective von Mises stress is twofold. Firstly, the elements with low stress values generally contribute larger errors to the cost function, due to low signal-to-noise ratio. Secondly, scaling by an element’s effective von Mises stress minimises the influence of the early stress–strain history and is therefore a practical way to reduce the importance of areas in the specimen which have not yielded. In the implementation by Pierron et al. [19], the stiffness-based optimised virtual fields were projected onto constant strain triangular elements. In this study the method was extended to quadrilateral elements so that the same type of virtual elements could be implemented for both the sensitivity and stiffness-based virtual fields. A different method must be used to calculate $\varvec{D}^{ep}$ since kinematic hardening is not considered here. The formulation for the elasto-plastic tangent stiffness matrix presented by de Souza et al. [16] was used. In order to integrate the element stiffness matrix, both the elasto-plastic stiffness matrix and the effective von Mises stress were averaged across each virtual element. The mean value for each was assigned to the centroid of the virtual element. The definitions of the modified global stiffness matrix and the tangential elasto-plastic stiffness matrix needed to compute the stiffness-based virtual fields are given in “Appendix 2”. This approximation of the element stiffness matrix limits how coarse the virtual mesh can be; the larger an elements is, the more points of measurement it contains as well as the larger area it spans. As a result the value at the centroid is estimated with larger error leading to reduction in effectiveness of noise-optimisation.

**Fig. 1:**
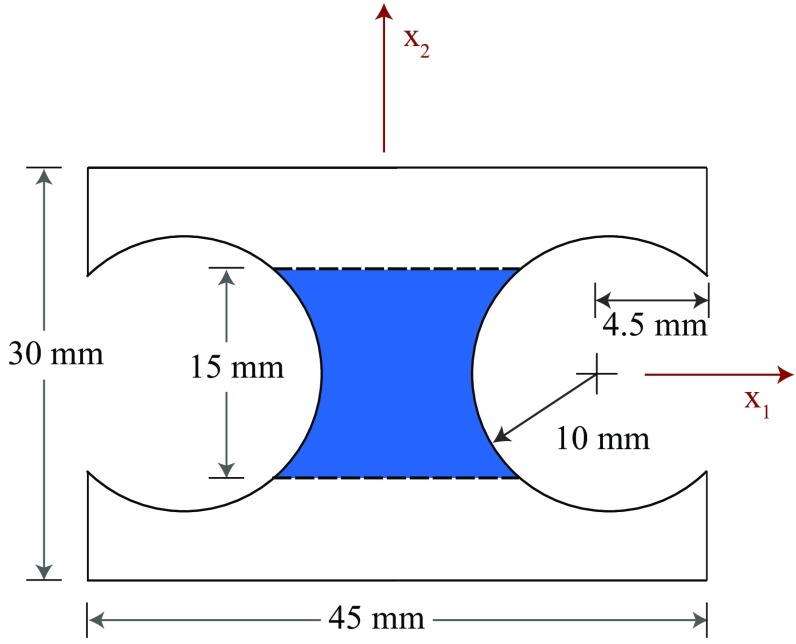
Geometry for the tensile specimen. The *blue* region in the centre of the specimen is the region of interest (ROI). (Color figure online)

## Application to simulated test data

To verify the effectiveness of the senstivity-based virtual fields, the method was tested using simulated data from uniaxial tensile test on a double-notched specimen. During the initial portion of the test, the material deforms elastically and no information on the yield or hardening response can be identified. Once the material yields a plastic zone is created. The parameters which define the yield behaviour of the material will be active on the boundary of this growing plastic zone. In the interior of the plastic zone, the parameters which describe the hardening response will be active. Therefore, the proposed sensitivity-based virtual fields for yield and hardening should follow the boundary and interior of the plastic zone, respectively. This can be used as a qualitative check that the sensitivity-based virtual fields have been correctly implemented.

### Double-notched tensile test

The simulated double-notched tensile test data was created to mimic a steel specimen that was subjected to an average longitudinal strain of 1%. The double notched specimen geometry pictured in Fig. 1 was selected because the deep notches produce a heterogeneous strain distribution. Similar geometry has already been used in [19]. The specimen was meshed in ABAQUS (v. 6.13) using 8-node bi-quadratic plane stress quadrilaterals (CPS8) with a total of 12,120 elements. The mesh density was chosen based on a mesh convergence study. The nodes on the bottom edge of the mesh were fixed and a vertical displacement of 0.3 mm was applied to the nodes on the top edge. The loading was imposed in 100 equal steps of 0.003 mm each. As a result, 100 different displacement fields are available for the identification process, simulating the recording of one hundred images during an experiment.

**Table 1:** Reference parameters for the linear and Voce hardening laws

	$\sigma _{0}$ (MPa)	*H* (MPa)	$R_{0}$ (MPa)	$R_{{inf}}$ (MPa)	*b*
Linear	297.5	3170	–	–	–
Voce	179.8	–	3170	117.7	3500

Two different constitutive models were considered. For both cases, the material model used linear elasticity ($E = 199\,\hbox {GPa}, \nu = 0.32$) combined with the von Mises yield criterion. Two different hardening laws were implemented: linear (Eq. 11) and Voce (Eq. 12). For a material that linearly hardens, the updated yield stress, $\sigma _y$, is a function of the equivalent plastic strain, $\bar{\varepsilon }^{p}$, the initial yield stress, $\sigma _0$, and the hardening modulus, *H*.

11 $$\begin{aligned} \sigma _y = \sigma _0 + H\bar{\varepsilon }^{p} \end{aligned}$$

A modified form of the Voce hardening law [19] was also implemented to include a non-linear hardening response. In this case, the updated yield stress is a function of the equivalent plastic strain and four model parameters: $\sigma _0$, the initial yield stress, $R_0$, the linear hardening modulus, and $R_{\mathrm {inf}}$ and *b* which describe the non-linear response at yield.

12 $$\begin{aligned} \sigma _y = \sigma _0 + R_{{inf}}\left( 1-\exp (-b\bar{\varepsilon }^{p})\right) +R_{0}\bar{\varepsilon }^{p} \end{aligned}$$

**Fig. 2:**
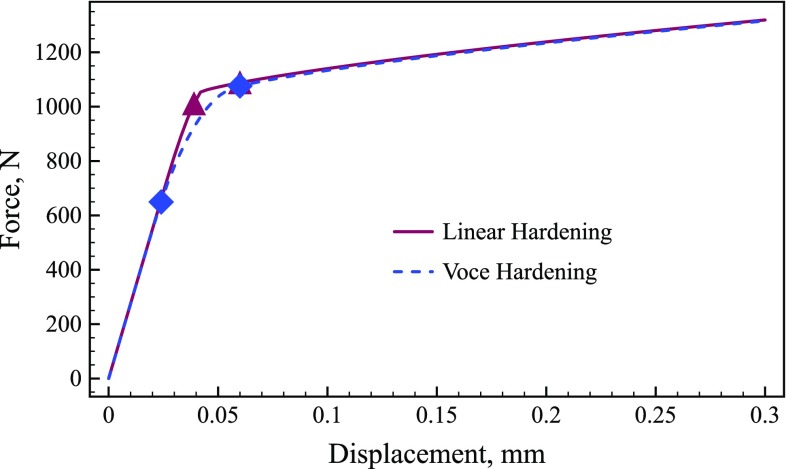
Force versus displacement curve for the linear and Voce hardening laws. The points highlighted on the *curve* for linear hardening correspond to time steps 13 and 20. The points highlighted on the *curve* for Voce hardening correspond to time steps 8 and 20

The parameters implemented in the FE model for both hardening laws are presented in Table 1. The values for the Voce law were based on the values cited in [19], whereas the linear hardening model was defined in such a way that it produces the same stress–strain relation as the Voce law as the plastic strain approaches infinity. The force-displacement curve for both hardening models is shown in Fig. 2.

### Simulated experimental data

To simulate the data that would be collected during an actual experiment, the resultant force at the top surface and the strain at the centroid of each element were exported from ABAQUS for each loading step. The strain data was interpolated onto a regular $150 \times 150$ mesh, covering the central portion of the specimen (Fig. 1) using the MatLab function *griddata* with a linear interpolant to simulate the format of data obtained from a typical full-field measurement technique such as DIC. Gaussian noise with a standard deviation of 150 $\mu \epsilon$ was artificially added to the simulated strain data to attempt to simulate data collected during an actual experiment. The white (Gaussian) noise was generated in MatLab using the function *randn*. The use of noisy data is necessary to evaluate the ability of the various virtual fields to minimize the effect of experimental noise on the parameter identification. This is thought to be enough to discriminate between the performances of the different virtual fields. Though beyond the scope of the present article, a more robust simulation of experimental data could be undertaken using image deformation [24]. This will be attempted in future work.

**Fig. 3:**
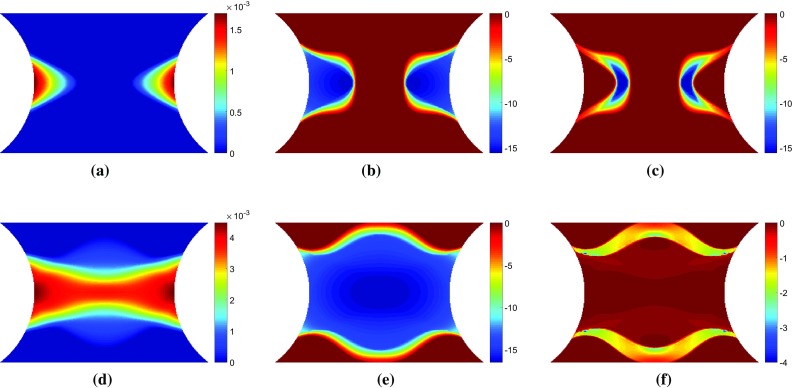
Maps of **a, d** equivalent plastic strain, **b, e** the vertical component of stress sensitivity to the yield stress, $\delta \sigma ^{(\sigma 0)}_{22}$, and **c, f** the incremental stress sensitivity, $\delta \tilde{\sigma }^{(\sigma 0)}_{22}$. The top row of maps **a**–**c** are for a displacement of 0.039 mm which corresponds to a resultant vertical force of 1011 N. The lower row of maps **d**–**f** are for a displacement of 0.060 mm which corresponds to a resultant vertical force of 1085 N

**Fig. 4:**
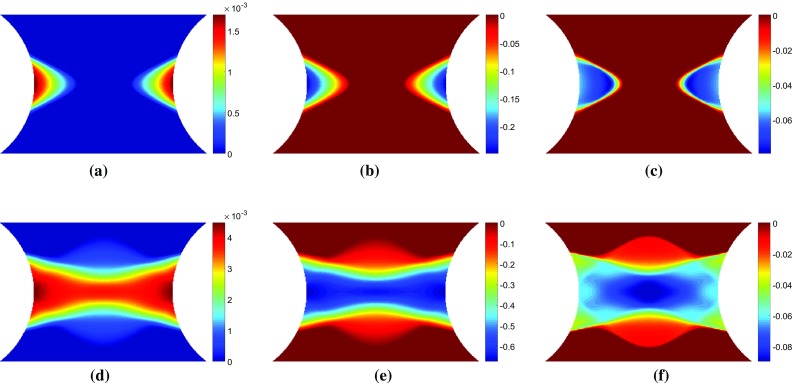
Maps of **a, d** equivalent plastic strain, **b, e** the vertical component of stress sensitivity to the hardening modulus, $\delta \sigma ^{(H)}_{22}$, and **c, f** the incremental stress sensitivity, $\delta \tilde{\sigma }^{(H)}_{22}$. The top row of maps **a**–**c** are for a displacement of 0.039 mm which corresponds to a resultant vertical force of 1011 N. The lower row of maps **d**–**f** are for a displacement of 0.060 mm which corresponds to a resultant vertical force of 1085 N

## Results and discussion

### Construction of the sensitivity-based virtual fields

To investigate whether the mathematical formulation presented in Sect. 3.1 highlights the regions where each parameter is active, maps of the equivalent plastic strain, $\bar{\varepsilon }^{p}$, the stress sensitivity, ${\delta \varvec{\sigma }}^{(i)}$, and the incremental stress sensitivity, ${\delta \widetilde{\varvec{\sigma }}}^{(i)}$, were drawn for both the linear and Voce hardening models. The stress sensitivity and incremental stress sensitivity were calculated using Eqs. 5–6 with exact data from their respective finite element models and the reference parameters given in Table 1. The small variation applied to the parameters in Eq. 5 was $\delta X_{i}=-0.05 X_{i}$. Here, the backwards finite difference was used in order to include slightly more points on the elasto-plastic boundary, compared to the forward finite difference.

**Fig. 5:**
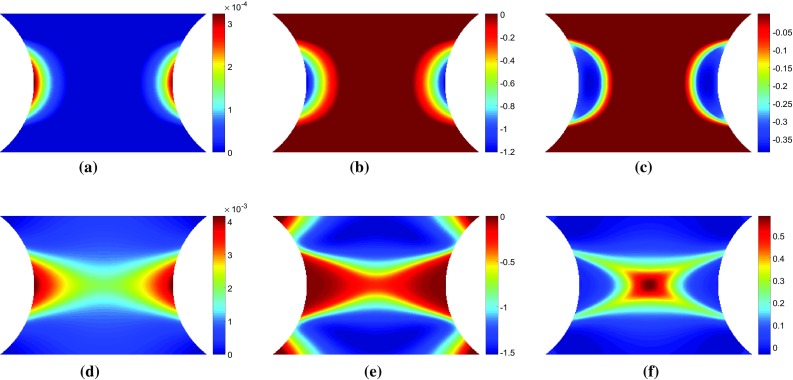
Maps of **a, d** equivalent plastic strain, **b, e** the vertical component of stress sensitivity to **b**, $\delta \sigma ^{(b)}_{22}$, and **c, f** the incremental stress sensitivity, $\delta \tilde{\sigma }^{(b)}_{22}$. The top row of maps **a**–**c** are for a displacement of 0.024 mm which corresponds to a resultant vertical force of 650 N. The lower row of maps **d**–**f** are for a displacement of 0.060 mm which corresponds to a resultant vertical force of 1076 N

#### Linear hardening

In Figs. 3 and 4, the maps of $\bar{\varepsilon }^{p}$, ${\delta \sigma _{22}}^{(i)}$, and ${\delta \widetilde{\sigma }_{22}}^{(i)}$ are shown for the linear hardening model at two different load steps. Videos of ${\delta \sigma _{22}}^{(i)}$ and ${\delta \widetilde{\sigma }_{22}}^{(i)}$ for all 100 time steps can be found in the Online Resources 1–2 for the yield stress and hardening modulus, respectively. The two time steps presented in Figs. 3 and 4 show the two main phases in the experiment: initial yielding (time step 13) and hardening of the entire centre region (time step 20). For reference these two time points are also marked in Fig. 2. In the first phase, the plastic zone propagates from the notches towards the centre of the specimen. In the second phase, the yield zone propagates from the centre towards the top and bottom of the specimen. As shown in Fig. 3b, e the stress sensitivity highlights the plastic zone, very closely resembling the shape of the equivalent plastic strain (Fig. 3a, d). The incremental stress sensitivity for yield stress (Fig. 3c, f) follows the boundary of the equivalent plastic strain, defining the border between the elastic and plastic zones, as expected. The incremental stress sensitivity focuses only on the regions that have yielded between the two increments, removing the history dependent effects shown in the stress sensitivity. However, Fig. 4 indicates that there is not much difference in shape between the stress sensitivity and incremental stress sensitivity for the hardening modulus. The only difference is the locations within the map that are emphasised; the magnitude of the stress sensitivity is the greatest where the equivalent plastic strain is the largest. The incremental stress sensitivity is the highest just behind the border of the plastic zone due to the removal of the history dependence. These results confirm that the incremental stress sensitivity, ${\delta \widetilde{\varvec{\sigma }}}^{(i)}$, highlights the regions in the specimen where each parameter is active.

**Fig. 6:**
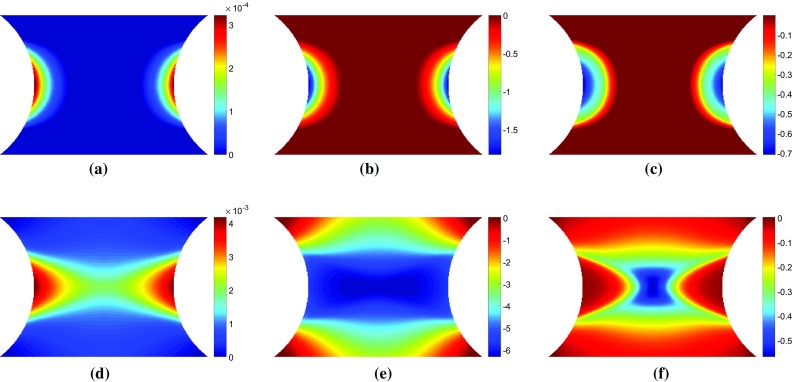
Maps of **a, d** equivalent plastic strain, **b, e** the vertical component of stress sensitivity to hardening modulus, $\delta \sigma ^{(R_{inf})}_{22}$, and **c, f** the incremental stress sensitivity, $\delta \tilde{\sigma }^{(R_{inf})}_{22}$. The top row of maps **a**–**c** are for a displacement of 0.024 mm which corresponds to a resultant vertical force of 650 N. The lower row of maps **d**–**f** are for a displacement of 0.060 mm which corresponds to a resultant vertical force of 1076 N

#### Voce hardening

The stress sensitivity and incremental stress sensitivity were also examined for the Voce model. As expected, the Voce parameters, $\sigma _{0}$ and $R_{0}$, behave almost exactly as the linear hardening parameters $\sigma _{0}$ and *H*. The remaining two parameters $R_{\mathrm {inf}}$ and *b* which capture the non-linear yielding response behave quite differently. Videos of the stress sensitivity and incremental sensitivity for all four model parameters are included in the Online Resources 3–6. The maps of both stress sensitivity and incremental sensitivity at two different time steps are presented in Figs. 5 and 6 for $R_{{inf}}$ and *b*, respectively. The two time steps correspond again to the propagation of the plastic zone from the notches (a–c) and the merging of the two plastic zones in the centre followed by vertical propagation (d–f). These two time steps are also marked in Fig. 2. At the onset of yielding, the stress sensitivity and incremental stress sensitivity for $R_{\mathrm {inf}}$ and *b* follow the boundary of the plastic zone (Figs. 5, 6a–c). As the plastic zone develops, clear differences between the stress sensitivity and incremental stress sensitivity emerge (Figs. 5, 6e–f). The incremental stress sensitivity for both parameters concentrates in the centre of the specimen, excluding the notched areas where plastic strain is the highest. The large differences in shape between the stress sensitivity and the incremental stress sensitivity are again due to the removal of history dependent effects by the incremental stress sensitivity. As the sample continues to plasticize the incremental stress sensitivities for $R_{\mathrm {inf}}$ and *b* occupy less space. This occurs because the exponential term in Eq. 12, which includes $R_{\mathrm {inf}}$ and *b*, decays to zero for large values of plastic strain. The stress sensitivity clearly highlights areas which are sensitive to a small change in the constitutive parameter but due to the intrinsic history-dependence of plasticity, any region where the parameter was active will still be highlighted. The incremental stress sensitivity effectively filters the history-dependence creating virtual fields that will follow the critical regions through time.

**Fig. 7:**
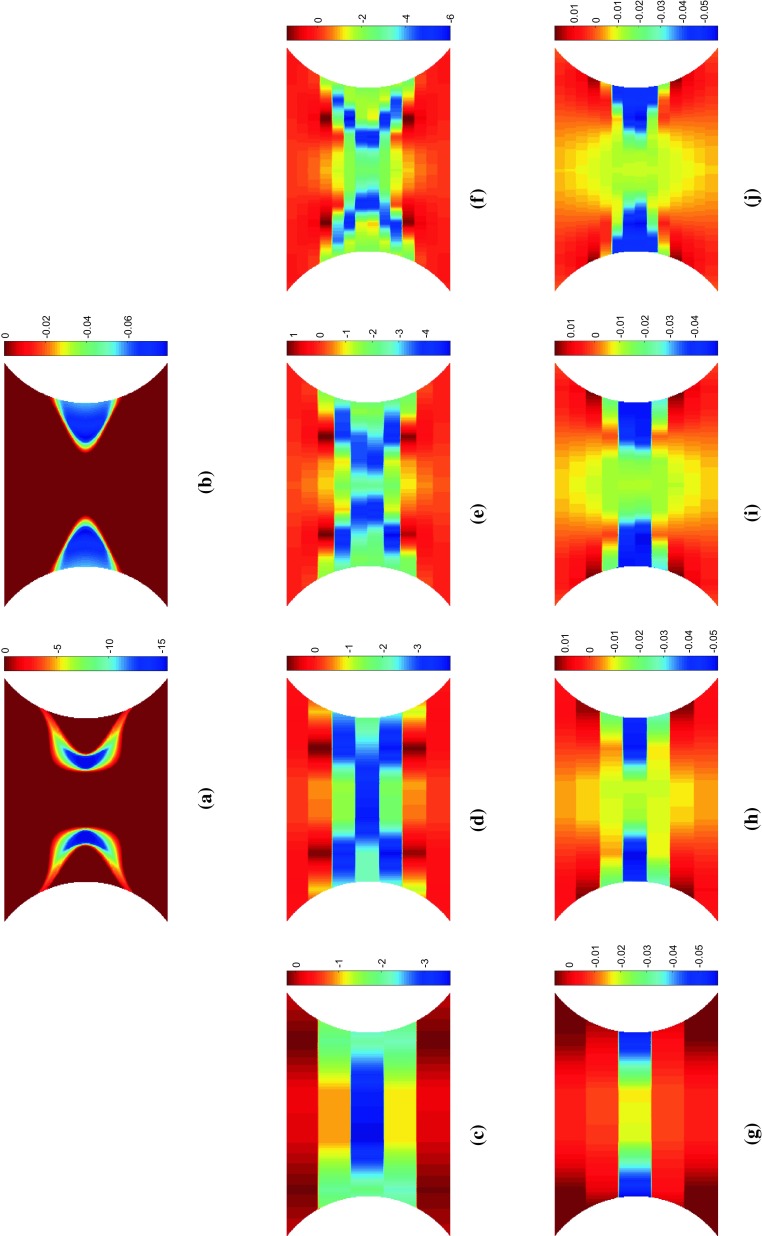
Maps of the sensitivity based optimised virtual fields (virtual strains) for the linear hardening model for different virtual mesh sizes: **c**, **g** $5 \times 5$, **d**, **h** $7 \times 7$, **e**, **i** $10 \times 10$ and **f**, **j** $14 \times 14$. For comparison, **a**–**b** show the incremental stress sensitivities, ${\delta \widetilde{\sigma }_{22}^{(\sigma _0)}}$ and ${\delta \widetilde{\sigma }_{22}^{(H)}}$, respectively

**Fig. 8:**
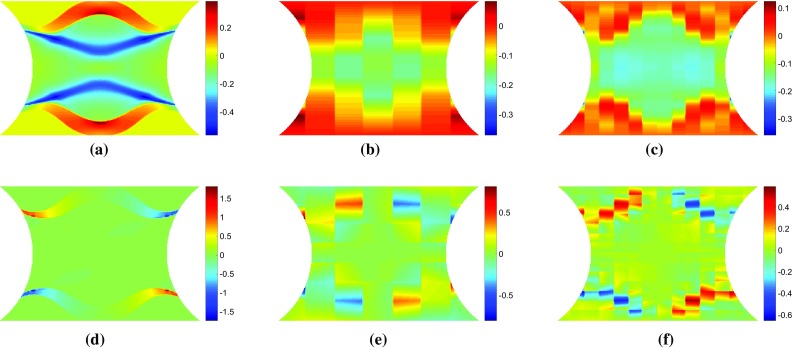
Maps of **a** ${\delta \widetilde{\sigma }_{11}^{(\sigma _0)}}$ and **c** ${\delta \widetilde{\sigma }_{12}^{(\sigma _0)}}$ the incremental stress sensitivity to the yield stress for the linear hardening model. The calculated virtual strains using the sensitivity based virtual fields **b**–**c** $\varepsilon _{11}^*$ and **e**–**f** $\varepsilon _{12}^*$ for using a (b,e) $7 \times 7$ and **c**, **f** $14 \times 14$ virtual mesh. The maps correspond to a vertical displacement of 0.060 mm and a resultant force of 1085 N

#### Sensitivity-based virtual fields

Sensitivity-based virtual fields were identified from the incremental stress sensitivity maps, ${\delta \widetilde{\varvec{\sigma }}}^{(i)}$, using Eq. 7. The virtual fields $\varepsilon _{22}^*$ that correspond to the incremental stress sensitivities in Figs. 3 and 4c, f are shown in Fig. 7. The piecewise linear functions are capable of reproducing the general spatial features of the stress sensitivity maps. It is worth noting that the virtual fields do not have to follow the sensitivity maps very precisely, but it is sufficient to highlight areas where the signal is present for each parameter at a particular time step. Due to some high gradients in the incremental stress sensitivity maps, the coarse mesh (Fig. 7c, g) struggles to capture these local variations. As the virtual mesh is refined, the features are more accurately reproduced as shown in Fig. 7d–f and h–j. The primary cost of mesh refinement is the computational time required to produce the global strain-displacement matrix, $\mathbf {B}$, which is needed to calculate the sensitivity-based virtual fields (Eq. 7). It is worth noting that the exact shape is not required for the method to be successful, as will be shown later; once the general shape is captured ($7 \times 7$ mesh) the virtual mesh has sufficient resolution to identify the model parameters for both the linear and Voce hardening models.

In Figs. 3 and 4, the stress sensitivity and incremental stress sensitivity were plotted in the loading direction. They can also be plotted for the remaining stress components. Figure 8a, d shows the incremental stress sensitivities, ${\delta \widetilde{\sigma }_{11}^{(\sigma _0)}}$ and ${\delta \widetilde{\sigma }_{12}^{(\sigma _0)}}$. The incremental stress sensitivity maps correspond to the second time step depicted in Figs. 3 and 4 when the yield zone propagates upwards and downwards from the specimen centre. The sensitivity-based virtual fields $\varepsilon _{11}^*$ and $\varepsilon _{12}^*$ are pictured in Fig. 8b, e and c, f for a $7 \times 7$ and $14 \times 14$ virtual mesh, respectively. The shapes of $\varepsilon _{11}^*$ and $\varepsilon _{12}^*$ are similar to their respective stress sensitivities but, the reconstructions are not as accurate as for $\varepsilon _{22}^*$. This mismatch occurs because the three virtual strain components are not independent. Since the incremental stress sensitivity in the loading direction is the largest in magnitude, the least-squares identification of the virtual strains places more weight on matching this component. Employing finer meshes improves the matching of the overall shapes for all three components but the method still struggles to match sharp gradients (e.g. pattern in Fig. 8d). The influence of the virtual mesh density on the parameter identification is further explored for each of the hardening models in Sects. 5.3.4 and 5.4.4.

**Fig. 9:**
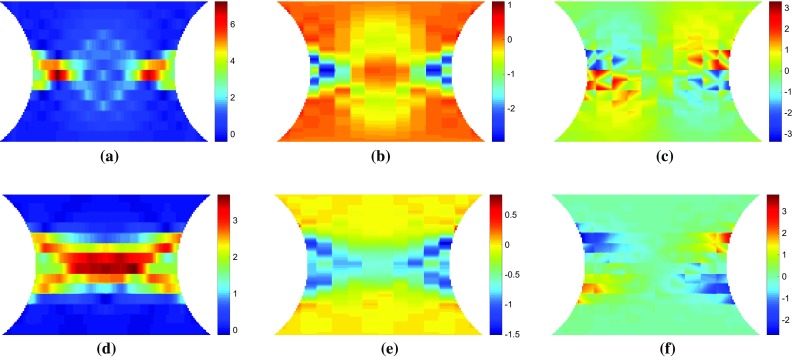
Virtual fields obtained for the stiffness-based optimised virtual fields ($14 \times 14$) for the linear hardening model at two different time steps. The *top row* of maps **a**–**c** are for displacement of 0.039 mm which corresponds to a resultant vertical force of 1011 N. The *lower row* of maps **d**–**f** are for a displacement of 0.060 mm which corresponds to a resultant vertical force of 1085 N

**Fig. 10:**
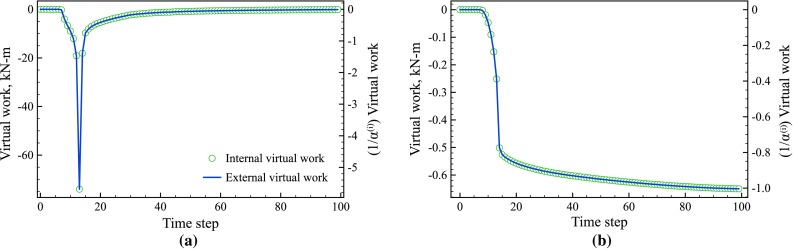
Evolution of internal ($\bigcirc$) and external (*blue line*) virtual work for the virtual fields for the **a** yield stress and **b** hardening modulus. The *y-axis* on the *left* is unscaled while the *y-axis* on the *right* has been scaled by $\alpha ^{(i)}$. (Color figure online)

### Construction of stiffness-based optimised virtual fields

The stiffness-based optimised virtual fields were found using the method described in “Appendix 2”. Only a single set of virtual fields, $\varepsilon ^* = [\varepsilon _{11}^{*}, \varepsilon _{22}^{*}, 2\varepsilon ^{*}_{12}]$, are calculated at each time step when using the stiffness-based virtual fields. This differs from the sensitivity-based procedure which produces a set of virtual fields for each model parameter. The stiffness-based virtual fields are shown in Fig. 9 for the linear hardening model and a $14 \times 14$ virtual mesh. The stiffness-based optimised virtual fields display a chequered pattern which is due to the instability of reduced integration bilinear quadrilateral elements. Employing full integration elements would remove the chequered pattern at the cost of interpolating the strain at the Gauss points. The full integration approach has been tested and the obtained results were consistent with the ones generated with the reduced integration approach, proving that the pattern does not have a detrimental influence on the identification process here. The shapes of the stiffness-based virtual fields, $\varepsilon _{11}^*$, appear to follow the shapes of the equivalent plastic strain maps (Fig. 3a, d). There does not appear to be any noticeable similarities between the sensitivity-based and stiffness-based virtual fields, indicating the each procedure focuses the identification on different regions.

### Validation on simulated data: linear hardening

The simulated data obtained from the finite element model was used to validate the approach for the linear hardening model. Firstly, the evolution of the internal and external virtual work with respect to time has been investigated to examine the time scale when each parameter is active. Secondly, the sensitivity-based, stiffness-based, and uniform virtual fields were used to identify the linear hardening parameters, $\sigma _0$ and *H*, from the simulated data. Then, noise was added to the strain data to simulate experimental conditions and all three kinds of virtual fields were again used to identify the two model parameters. Finally, the influence of the virtual mesh density on the identified parameters has been evaluated. In this study, the identification of elastic parameters has been ignored as it can be done using only elastic loading with the linear VFM [1], effectively reducing computational effort in minimising the cost function.

#### Evolution of the internal and external virtual work

To examine the time steps when each parameter is active, the internal virtual work was calculated for each parameter using Eq. 9. Figures 10a, b show the internal and external virtual work for the yield stress and hardening modulus, respectively. The magnitude and shape of the curves for the yield stress and hardening modulus are clearly different. The internal and external virtual work for both the yield stress and hardening modulus remain at zero for the first 9 steps corresponding to elastic loading, hence completely filtering out the elastic part of the test from the cost function. Then at time step 10, which corresponds to the onset of plasticity at the notches, the virtual work starts to increase from zero as expected. The internal virtual work for the yield stress quickly peaks at the 15$\mathrm {th}$ time step, which corresponds to the plastic zone spreading across the whole specimen and then decays back to zero. In contrast, the internal virtual work for the hardening modulus continuously increases throughout the test. The magnitude of the internal virtual work for the yield stress and hardening modulus are also markedly different. To properly identify the model parameters using Eq. 8, the virtual work is scaled by $\alpha ^{(i)}$ using the 15 highest IVW values to ensure that the contributions of each parameter to the cost function are of the same order. The y-axis on the right hand side of Fig. 10 shows the scaled values for both the internal and external virtual work. Although not backed by any physical argument, the scaling method employed here was proven to be successful as shown in Sect. 5.4.5, where it was found that the number of time steps taken for computing $\alpha ^{(i)}$ has minor effect on the identification errors.

#### Identification without noise

To identify the linear hardening parameters, $\sigma _0$ and *H*, the cost function given in Eq. 8 was minimized using the built in MatLab (v. 6.14b) function *fmincon* and the SQP (Sequential Quadratic Programming) algorithm. The model parameters were constrained to be greater than zero. This restriction was imposed to ensure that the material response was physically reasonable for the steel being studied. The initial guess supplied to the minimization function was generated with a random number generator. To verify that the set of identified material parameters represented a global minimum, 15 different starting points were tried. Since the same set of parameters were consistently obtained independent of the initial guess, the identified parameters were assumed to be the global minimum. The results obtained with the uniform, stiffness-based, and sensitivity-based virtual fields are presented in Table 2. $7 \times 7$ and $14 \times 14$ virtual meshes were used for the sensitivity-based and stiffness-based virtual fields, respectively; it should be noted that finer meshes are required for the stiffness-based virtual fields as described in Sect. 3.3. All methods accurately identified the model parameters since the principle of virtual work is satisfied exactly on perfect (noise-free) data. This verifies that all three virtual field types were implemented correctly, leading to an identification error smaller than 1$\%$ in Table 2. The virtual mesh density for the sensitivity-based virtual fields was varied from $5 \times 5$ to $14 \times 14$ and no change was observed in the identified model parameters.

**Table 2:** Identified parameters for the linear hardening model using exact data

	$\sigma _{0}/\sigma _{0}^{ref}$	$H/H^{ref}$
Uniform	1.003	0.994
Stiffness	0.998	0.998
Sensitivity	1.000	1.001

#### Identification with noise

The parameters for the linear hardening model were also identified using noisy data. Gaussian white noise with a standard deviation of 150 $\mu \epsilon$ was added to strain data obtained from the finite element simulation. This level of noise represents what is expected in a well designed and conducted experiment. It should be emphasized that this is only a first approach to noise propagation simulation. Recent studies [24, 27] have shown that an image deformation procedure needs to be employed to realistically simulate both systematic and random errors. However, this procedure is more computationally extensive and has so far only been applied to linear elasticity. This approach will be investigated in the future for elasto-plastic identification and a simpler noise study has been employed here. It is thought however that this simplified procedure will be enough to get a first idea about the relative stability of the different virtual fields to noise. As the radial-return algorithm employed here for stress calculation uses strain increments rather than total strain, it is worth comparing the magnitude of the noise to that of the average strain increment. For the strain in the loading direction, $\varepsilon _{22}$, the mean value of strain increment after yielding is $165~\mu \epsilon$, making the effective signal-to-noise ratio approximately 1.1. Effectively, such high noise can produce spurious elastic unloadings which were shown to heavily influence the cost function and amplify the identification error significantly [15]. In order to increase the signal-to-noise ratio, the number of time steps can be reduced by only using data from every *n* *th* time step to increase the strain increment between consecutive frames. This procedure effectively increases the signal-to-noise ratio (due to the rate nature of the plasticity equations). In practice, more images means that temporal smoothing can indeed be used to further increase the signal-to-noise ratio. Moreover, if too few steps are used, then the radial return algorithm will generate stress reconstruction errors so a compromise has to be found. In this paper four different total time steps were tested, 100, 50, 33, and 25, resulting in an effective signal-to-noise ratio of 1.1, 2.1, 3.2, and 4.3, respectively. Although increasing the strain increment can lead to errors in the radial return algorithm, using the simulated data there was found to be a less than 0.1% difference between the stresses predicted using 100 and 25 time steps. Similar to the case without noise, the identification was repeated 15 times and the same parameters were identified independently from the initial guesses. In order to estimate both random and systematic errors, 30 different copies of noise were added to the simulated data. The mean value and the coefficient of variation of the identified parameters for the uniform, stiffness-based, and sensitivity-based virtual fields are given in Table 3 where a virtual mesh size of $7 \times 7$ and $14 \times 14$ was implemented for the sensitivity-based and stiffness-based virtual fields, respectively.

**Table 3:** Identified parameters for linear hardening model using noisy data

	Time Steps	Signal-to-noise ratio	$\sigma _{0}/\sigma _{0}^{ref}$	$H/H^{ref}$
Uniform	100	1.1	$1.137 \pm 0.0076\%$	0
	50	2.1	$1.013 \pm 0.031\%$	$1.346 \pm 0.49\%$
	33	3.2	$1.022 \pm 0.024\%$	$0.967 \pm 0.23\%$
	25	4.3	$1.019 \pm 0.024\%$	$0.944 \pm 0.20\%$
Stiffness	100	1.1	$1.140 \pm 0.010\%$	0
	50	2.1	$0.987 \pm 0.041\%$	$1.351 \pm 0.52\%$
	33	3.2	$1.003 \pm 0.013\%$	$1.023 \pm 0.11\%$
	25	4.3	$1.004 \pm 0.015\%$	$1.003 \pm 0.085\%$
Sensitivity	100	1.1	$1.037 \pm 0.26\%$	$1.254 \pm 13\%$
	50	2.1	$1.022 \pm 0.073\%$	$0.999 \pm 1.3\%$
	33	3.2	$1.014 \pm 0.025\%$	$0.994 \pm 0.21\%$
	25	4.3	$1.012 \pm 0.027\%$	$0.978 \pm 0.21\%$

**Fig. 11:**
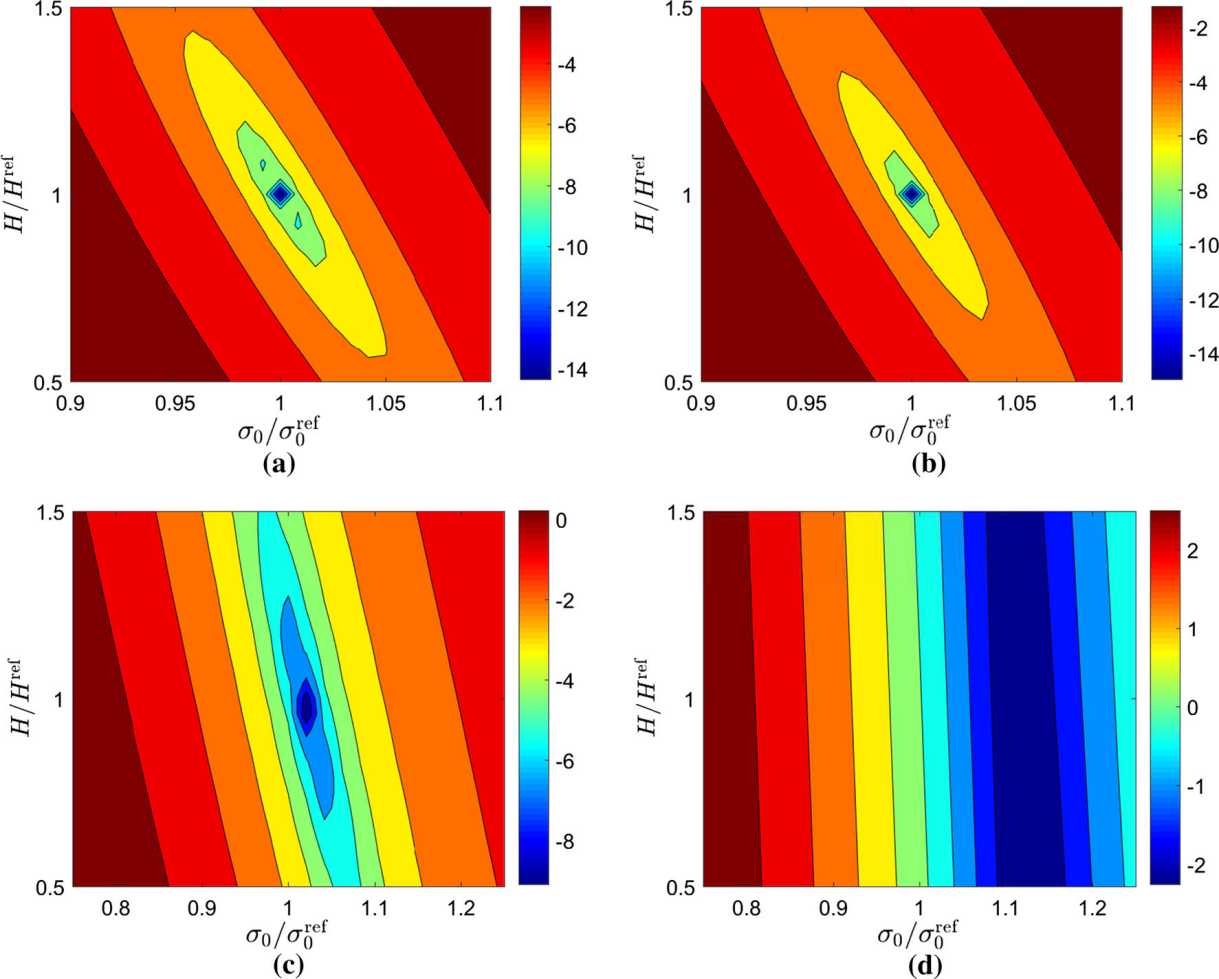
Plots of the cost functions obtained for the linear hardening model using Eq. 11 and uniform virtual fields (Eq. 10). The *upper row* **a**–**b** corresponds to **a** 33 time steps and **b** 100 time steps for perfect data. The *lower row* **c**–**d** corresponds to **c** 33 time steps and **d** 100 time steps for noisy data. Note that the log of the cost function is plotted to better illustrate the minimum

**Fig. 12:**
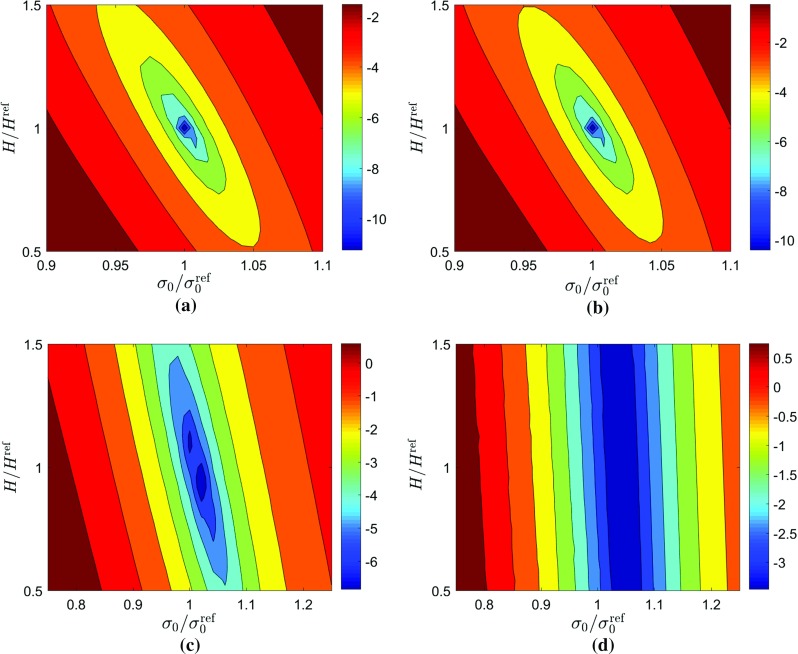
Plots of the cost functions obtained for the linear hardening model using Eq. 11 and sensitivity-based virtual fields. The *upper row* **a**–**b** corresponds to **a** 33 time steps and **b** 100 time steps for perfect data. The *lower row* **c**–**d** corresponds to **c** 33 time steps and **d** 100 time steps for noisy data. Note that the log of the cost function is plotted to better illustrate the minimum

As the signal-to-noise ratio of the strain increment increases with a smaller number of time steps, the accuracy of the identification increases for all methods. Decreasing from 33 to 25 time steps worsens the accuracy of the identification for the hardening modulus indicating that there is insufficient temporal resolution. Notably, from 50 time steps downwards, the yield stress is accurately identified with all methods. The sensitivity-based virtual fields are able to identify the hardening modulus using 50 steps, which corresponds to a signal-to-noise ratio of 2.1. However the other methods struggle to identify *H* at signal-to-noise levels below 3.2 (33 time steps). Using all 100 time steps, the lower bound of the optimization algorithm was reached for the uniform and stiffness-based virtual fields resulting in $H=0$. The zero value for *H* is compensated for by overestimated $\sigma _0$ values. As the signal-to-noise ratio increases, the random error consistently increases as well for every virtual field. The reduction in the number time steps likely causes this increase in random error; the reduction in time steps makes the identification mores susceptible to noise which results in increased random error.

The influence of noise on the cost function can be seen in Figs. 11 and 12 for the uniform virtual field. For exact data (Fig. 11a, b) there is a clear minimum which does not depend on the number of time steps used, supporting the fact that the identified value is the global minimum. When the signal-to-noise ratio is high (low number of time steps) the cost function is not appreciably changed by the addition of noise (Fig. 11a, c). For a smaller signal-to-noise ratio (Fig. 11d), a valley with little sensitivity to hardening modulus was formed. A similar behaviour is observed for the sensitivity-based virtual fields (Fig. 12), though the minimum for *H* is slightly closer to the expected value.

**Table 4:** Study of the influence of the mesh size on the identified parameters of the linear hardening law using sensitivity-based virtual fields

Virtual mesh	$\sigma _{y}/\sigma _{y}^{ref}$	$H/H^{ref}$
$5 \times 5$	$1.014 \pm 0.027\%$	$0.994 \pm 0.23\%$
$7 \times 7$	$1.014 \pm 0.025\%$	$0.994 \pm 0.21\%$
$10 \times 10$	$1.013 \pm 0.025\%$	$0.993 \pm 0.21\%$
$14 \times 14$	$1.013 \pm 0.023\%$	$0.992 \pm 0.19\%$

#### Sensitivity to the virtual mesh size

As described earlier, the size of the virtual mesh influences how well the virtual strains match the incremental stress sensitivity maps. To determine if the mesh density influences the identification results, three additional mesh densities were tested: $5 \times 5$, $10 \times 10$, and $14 \times 14$. The identification procedure was run 30 times, each time with a different copy of noise. The mean and coefficient of variation of the parameters are reported in Table 4. The mesh density does not have a significant influence on the mean value of *H* or $\sigma _0$. The coefficient of variation slightly decreases with increasing mesh density. The accurate identification of *H* and $\sigma _0$ also proves that the differences between the incremental stress sensitivity and the derived virtual fields observed in Figs. 8 and 7 do not impact the identification procedure. This is a very positive outcome as a strong virtual mesh sensitivity would have required mesh density optimization.

### Validation on simulated data: Voce hardening

**Fig. 13:**
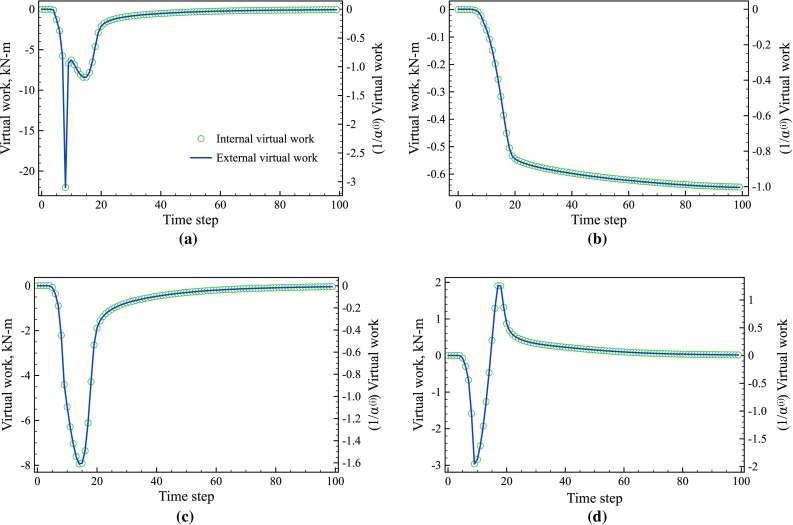
Evolution of internal ($\bigcirc$) and external (*blue line*) virtual work, respectively for the sensitivity-based virtual fields for **a** $\sigma _0$, **b** $R_0$, **c** $R_{\mathrm {inf}}$, and (d) *b*. The *y-axis* for each parameter has been scaled by $\alpha ^{(i)}$. (Color figure online)

The Voce hardening model was also considered to determine how increasing the complexity of the constitutive relationship would influence the inverse parameter identification. Based on the sensitivity study discussed at the end of this section, a virtual mesh of $14 \times 14$ was used and the perturbation and scaling parameters were fixed to $\delta X_{i}=-0.10X_{i}$ and 30% of the highest IVW terms (Eq. 9).

#### Evolution of the internal and external virtual work

The internal virtual work (Eq. 9) was used to identify when each parameter was active (Fig. 13). The three parameters, $\sigma _{0}$, $R_{\mathrm {inf}}$, and *b*, that describe the onset of yielding were active from time steps 7 to 30. The hardening parameter, $R_0$, also became active at time step 7 but continued to grow until the test ended. The graphs in Fig. 13 have two y-axes, the left-hand axis shows the unscaled values while the right-hand axis has been scaled by $\alpha ^{(i)}$ which is the mean of the 30% highest values of the IVW terms.

#### Identification without noise

The parameters were identified using the same procedure outlined for linear hardening in Sect. 5.3.2. Again all of the model parameters were constrained to be positive. In addition, the hardening parameter, $R_{0}$, was constrained such that $R_0 \ge 1000$ MPa in order to narrow the search region. The results obtained for all types of virtual fields, with the same $14 \times 14$ virtual mesh for both the sensitivity-based and stiffness-based virtual fields, are shown in Table 5. All of the methods are clearly capable of identifying the model parameters well. It should be noted that parameters $\sigma _{0}$ and $R_{\mathrm {inf}}$ are distinguishable only at the onset of plasticity. After some plastic deformation is accumulated, it is the combined value, $Y = \sigma _{0} + R_{\mathrm {inf}}$, that influences the cost function. This enables the values of $\sigma _{0}$ and $R_{\mathrm {inf}}$ to compensate for one another and as a result, the simulated data mainly contains information about *Y* and limited data about $\sigma _{0}$ and $R_{\mathrm {inf}}$ individually.

**Table 5:** Identified parameters of the Voce hardening model using exact data

	$\sigma _{0}/\sigma _{0}^{ref}$	$R_{0}/R_{0}^{ref}$	$R_{\mathrm {inf}}/ R_{\mathrm {inf}}^{ref}$	$b/b^{ref}$	$Y/Y^{ref}$
Uniform	1.001	1.000	1.000	1.000	1.000
Stiffness	1.002	1.002	0.997	1.008	1.000
Sensitivity	1.002	1.000	0.998	0.997	1.001

**Fig. 14:**
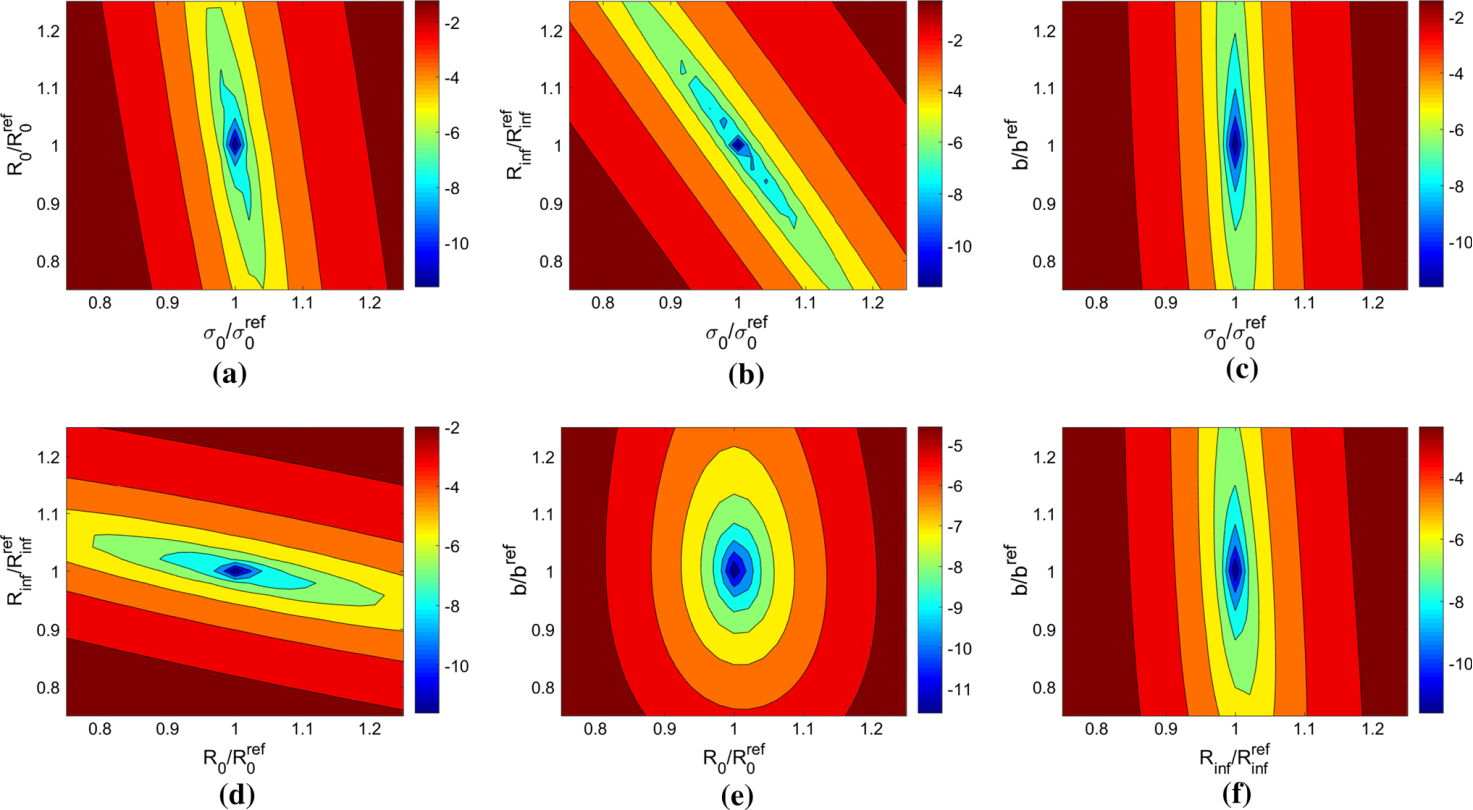
Cuts through a cost function for Voce model with sensitivity-based virtual fields obtained with exact data and 33 time steps. The *plots* show interaction between pairs of parameters: **a** $\sigma _{0}-R_{0}$, **b** $\sigma _{0}-R_{{inf}}$, **c** $\sigma _{0}-b$, **d** $R_{0}-R_{{inf}}$, **e** $R_{0}-b$, (f) $R_{inf}-b$. Note that the log of the cost function is *plotted* to better illustrate the minimum

The cost function for the Voce model with exact data is shown in Fig. 14. Since this model has 4 parameters, the cost function is situated in a 4-dimensional space. To visualize the cost function several planes were cut through the cost function. In Fig. 14, six plots showing the interaction between pairs of parameters are reported. In each case, an elongated locus with a unique minimum is observed. The plot of $\sigma _{0}$ vs $R_{inf}$ (Fig. 14b) indicates that there is a strong correlation between the two parameters, represented by the open valley inclined at an angle close to $45^{\circ }$. Both $\sigma _{0}$ and $R_{inf}$ show minimal dependence on *b* as shown in Fig. 14c, f with open valleys aligned with reference values of the yielding parameters. There is however a very well defined minimum on the $R_{0}-b$ cut (Fig. 14e), suggesting that the *b* parameter is found because of this interaction.

**Table 6:** Identified parameters for the Voce hardening model using noisy data

Virtual fields	Time steps	$\sigma _{0}/\sigma _{0}^{ref}$	$R_{0}/R_{0}^{ref}$	$R_{inf}/R_{inf}^{ref}$	$b/b^{ref}$	$Y/Y^{ref}$
Uniform	50	$1.218 \pm 0.35\%$	$0.319 \pm 1.8\%$	$1.141 \pm 1.1\%$	$0.332 \pm 0.32\%$	$1.188 \pm 0.27\%$
	33	$1.252 \pm 0.29\%$	$0.316 \pm 0$	$0.988 \pm 0.47\%$	$0.287 \pm$ 0.23$\%$	$1.148 \pm 0.021\%$
	25	$1.234 \pm 0.47\%$	$0.470 \pm 0.78\%$	$0.921 \pm 0.83\%$	$0.328 \pm 0.45\%$	$1.110 \pm 0.12\%$
Stiffness	50	$0.954 \pm 0.76\%$	$1.371 \pm 0.34\%$	$1.035 \pm 1.2\%$	$1.545 \pm 1.4\%$	$0.986 \pm 0.027\%$
	33	$1.072 \pm 0.30\%$	$1.024 \pm 0.10\%$	$0.905 \pm 0.44\%$	$0.971 \pm 0.88\%$	$1.006 \pm 0.020\%$
	25	$1.076 \pm 0.59\%$	$1.007 \pm 0.059\%$	$0.897 \pm 0.86\%$	$0.948 \pm 1.0\%$	$1.005 \pm 0.020\%$
(Incremental) sensitivity	50	$1.102 \pm 2.2\%$	$1.070 \pm 7.3\%$	$0.893 \pm 2.7\%$	$0.949 \pm 8.1\%$	$1.019 \pm 0.54\%$
	33	$1.060 \pm 3.1\%$	$1.012 \pm 2.9\%$	$0.939 \pm 4.0\%$	$0.976 \pm 9.0\%$	$1.013 \pm 0.34\%$
	25	$1.054 \pm 0.77\%$	$1.005 \pm 0.26\%$	$0.941 \pm 1.2\%$	$0.968 \pm 1.4\%$	$1.009 \pm 0.037\%$
Stress sensitivity	33	$1.377 \pm 2.4 \%$	$0.921 \pm 9.0 \%$	$0.501 \pm 0.33 \%$	$1.563 \pm 57\%$	$1.031 \pm 1.4\%$

#### Identification with noise

To test the ability of the different virtual fields to minimize the influence of noise, Gaussian noise with a standard deviation of 150 $\mu \epsilon$ was added to the finite element strains. Based on the identification of the linear hardening parameters, only 50, 33, and 25 time steps were used to perform the identification resulting in a signal-to-noise ratio of approximately 2.2, 3.3, and 4.4, respectively. The minimization program was run 15 times varying the initial guess. This time, the different starting points resulted in different sets of identified parameters, so the set of parameters that produced the lowest value of the cost function was taken as the global minimum. This process was repeated 30 times with different copies of noise. Thus far, the incremental stress sensitivity maps have been used to generate the virtual fields, however it is also possible to use Eq. 5 to construct the stress-sensitivity virtual fields. The mean and coefficient of variation of the parameters identified using the noisy data are given in Table 6. It should be noted that for the stress-sensitivity virtual fields only 33 time steps were used.

For the uniform virtual field, the lower bound for $R_0$ was frequently reached for 50 and 33 time steps ($R_{0}/R_{0}^{ref} = 1000/3170 = 0.316$). In fact, the coefficient of variation of $R_0$ for 33 time steps is zero because the lower bound of the minimization routine was reached with every copy of noise. The sensitivity-based and stiffness-based virtual fields clearly out-perform the uniform virtual fields, most notably for $R_0$ and *b*. For the more complex Voce model, the uniform virtual fields struggled to identify the yield stress, $\sigma _0$, linear hardening modulus, $R_0$, and the non-linear yield parameter, *b* even for high signal-to-noise ratios. The stress-sensitivity virtual fields significantly overestimate $\sigma _{0}$ and *b* and underestimate of $R_{inf}$. However the hardening modulus, $R_0$, is well identified. Since the history dependence has not been removed from the stress-sensitivity virtual fields, the later stages of the test are given a much larger weight and as a result, the stress-sensitivity virtual fields do not identify the yielding parameters well.

**Fig. 15:**
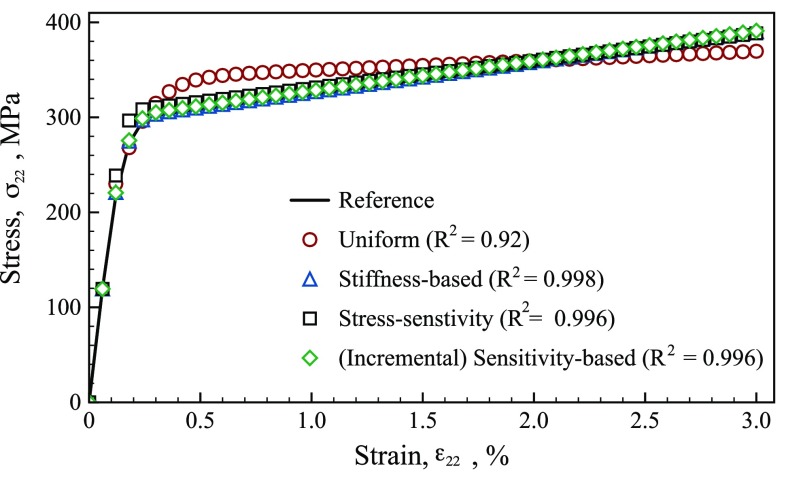
Stress–strain curve obtained with parameters identified in Table 6 for 33 time steps

To visualize the difference between the parameters in Table 6, the stress–strain curves that would be obtained using the parameters for 33 time steps are shown in Fig. 15. The parameters identified using the sensitivity-based, stress-sensitivity, and stiffness-based virtual fields all produce stress–strain curves that closely follow the reference curve ($\mathrm {R}^2 = 0.99$). In Table 6, the uniform and stress-sensitivity virtual fields over-estimate the yield stress and the higher yield stresses are clearly visible in Fig. 15. While for the uniform virtual field the overestimated yield stress was paired with an underestimated hardening modulus, the stress-sensitivity virtual fields overestimate the yield stress and capture the hardening response. However. this mis-identification of the model parameters for the stress-sensitivity virtual fields does not appear to impair the ability to follow the reference stress–strain curve. In fact, except at the onset of yield; the stress-sensitivity virtual fields generated data, closely follow the incremental stress-sensitivity one.

**Fig. 16:**
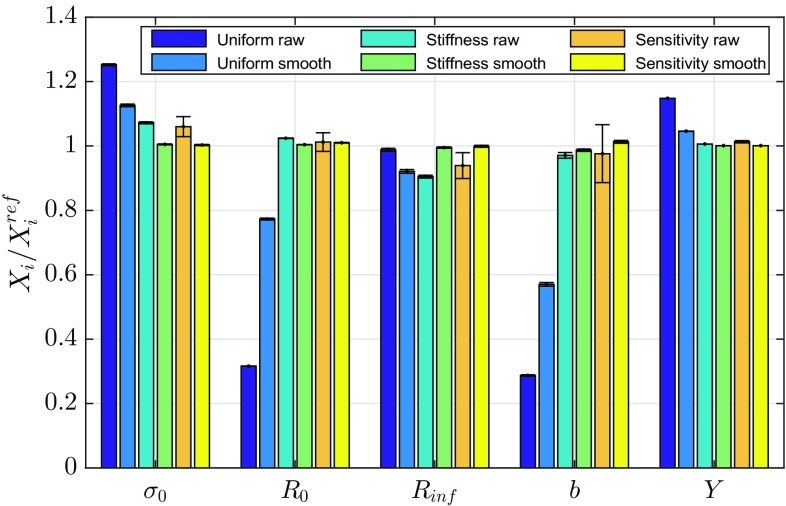
Comparison of mean values of identified parameters using different VFs over 30 copies of noise. The uncertainty *bars* represents one standard deviation

Typically, to minimize the influence of noise, some form of temporal smoothing would be used on the measured displacement, especially when plasticity occurs since the noise can cause spurious elastic unloading [15]. To determine what effect temporal smoothing would have on the identification, the full data set using all 100 points was smoothed with a simple moving average over a window of five data points. The data was then reduced to 33 points by keeping only every third smoothed data point. Using this temporally smoothed data set, the identification was repeated 30 times to determine the mean and standard deviation of the identified parameters. In Fig. 16, the results for data that has been temporally smoothed is compared with unsmoothed noisy data. The identification using the uniform virtual fields is improved significantly. The parameters for the stiffness-based and sensitivity-based virtual fields are all within 1.5% of the reference values.

#### Sensitivity to the virtual mesh size

To ensure that the mesh density had a minimal impact on the identification, three additional virtual mesh densities were tested: $5 \times 5$, $7 \times 7$, and $10 \times 10$. Table 7 reports the mean and coefficient of variation obtained for each of the parameters when the identification was run 30 times with different copies of noise. Similar to the results obtained for the linear hardening model, the mesh density did not affect the mean value of the identified parameters but increasing the mesh density tended to cause the random error to slightly decrease.

**Table 7:** Influence of virtual mesh size on identified parameters for Voce model

Mesh	$\sigma _{0}/\sigma _{0}^{ref}$	$R_{0}/R_{0}^{ref}$	$R_{inf}/R_{inf}^{ref}$	$b/b^{ref}$	$Y/Y^{ref}$
$5 \times 5$	$1.094 \pm 4.0\%$	$1.064 \pm 4.7\%$	$0.887 \pm 5.3\%$	$0.920 \pm 13\%$	$1.012 \pm 0.60\%$
$7 \times 7$	$1.109 \pm 3.8\%$	$1.042 \pm 5.0\%$	$0.864 \pm 5.4\%$	$0.873 \pm 11\%$	$1.012 \pm 0.60\%$
$10 \times 10$	$1.089 \pm 3.3\%$	$1.042 \pm 3.0\%$	$0.889 \pm 4.4\%$	$0.935 \pm 11\%$	$1.010 \pm 0.40\%$
$14 \times 14$	$1.090 \pm 2.8\%$	$1.041 \pm 3.6\%$	$0.886 \pm 3.9\%$	$0.926 \pm 7.2\%$	$1.009 \pm 0.40\%$

**Fig. 17:**
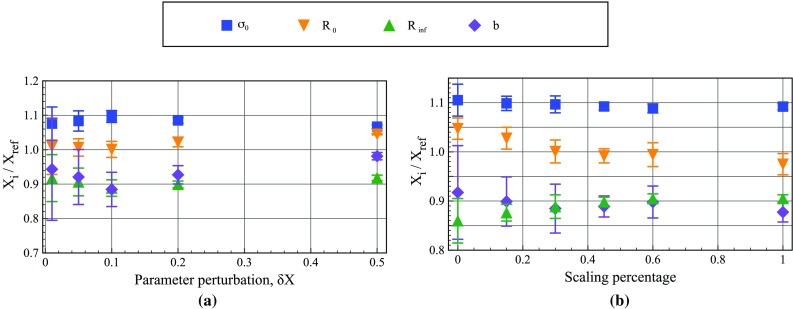
Relative error on the identification versus the implemented **a** perturbation parameter and **b** scaling percentage. The *error bars* show one standard deviation

#### Sensitivity to reconstruction parameters

The proposed method includes two parameters that must be selected, the perturbation, $\delta X_i$, and the scaling parameter, $\alpha ^{(i)}$. The perturbation will influence the stress sensitivity and its reconstruction while the scaling will directly influence the cost function (Eq. 8). For the Voce model results, the perturbation and scaling parameters were fixed to $\delta X_{i}=-0.10X_{i}$ and 30% of the highest IVW terms (Eq. 9), respectively. The effect of both these parameters on the quality of identification was studied on a noisy data set with 33 time steps. The mean and standard deviation for the four model parameters were identified by running the identification 30 times with different copies of noise.

To study the influence of the perturbation, $\delta X_{i}$, a virtual mesh of $14 \times 14$ and a scaling parameter of 30% were used. A fine mesh was implemented because smaller virtual elements are needed to capture the fine features and high gradients that would be produced as the perturbation shrinks. Five different values were tested: 0.01, 0.05, 0.1, 0.2 and 0.5. In Fig. 17a the results are presented showing how the identified value of each model parameter varied as a function of the perturbation parameter. The random error on the identification drastically decreases as the perturbation parameter is increased. As the perturbation increases so does the error on the hardening modulus, $R_0$. The best compromise for the perturbation parameter appears to be 10%, after which the bias on $R_0$ increases. However, the sensitivity of this parameter is rather small, which is an important feature for the procedure as one would not want the results to be highly affected by the users choice of the perturbation parameter.

The full range of the scaling parameter, $\alpha ^{(i)}$, was also investigated using a virtual mesh of $14 \times 14$ and a perturbation of 0.10. For the two extreme cases of 0 and 100%, the cost function was scaled by the maximum and mean of the internal virtual work, respectively. In addition, 6 other scaling parameters were also tested, the mean of the highest 10, 15, 20, 30, 45, and 75% of the IVW terms. The results are shown in Fig. 17b. The scaling has a minimal effect on $\sigma _0$. The remaining parameters show modest changes of approximately 5% on the mean identified value with the largest variation observed in the hardening modulus, $R_0$. The optimum scaling parameter appears to be 45% where the random error and bias for all of the parameters is the lowest.

## Conclusions

In this manuscript, a new set of virtual fields for non-linear constitutive models has been proposed. These virtual fields are formed using incremental stress sensitivity maps to locate the areas and times when each constitutive parameter has the most impact on the stress. The feasibility of the sensitivity-based virtual fields was tested for small strain plasticity implementing two different hardening laws: linear and Voce. However, the sensitivity-based virtual fields could be broadly implemented for any non-linear constitutive model. The sensitivity-based virtual fields were consistently able to identify the plastic model parameters even for low signal-to-noise ratios (Tables 3 and 6), indicating their ability to smooth out the influence of noise on the parameter identification. While the incremental stress sensitivity virtual fields consistently performed well, the stress-sensitivity based virtual fields failed to accurately identify the parameters. The results obtained with the sensitivity-based virtual fields were also compared with stiffness-based and manually defined uniform virtual fields and the sensitivity-based virtual fields were found to outperform the two alternatives, even though the stiffness-based fields also showed good stability to noise.

The sensitivity-based virtual fields provide a general approach to automatically generate high quality virtual fields for non-linear VFM problems. An open question remains concerning the high random error exhibited by sensitivity-based virtual fields when raw data was used. One possible explanation is that the virtual fields select only ‘active’ zones in the specimen and filter out the remaining data which makes the identification very sensitive to the noise pattern. This possibility is further supported by observation that the random error drops drastically when the perturbation $\delta X$ is increased. For the yield-related parameters, the perturbation simply controls the width of the zone around the yield front. Nevertheless, in real experiment, some temporal smoothing would be introduced which reduces the random error to levels exhibited by the other virtual fields as shown in Fig. 16.

The implementation of the sensitivity-based virtual fields also has several limitations which need to be discussed. First, for the identification of the Voce parameters from noisy data, the minimisation routine did not always converge to the global minimum. By running the minimisation 15 times with different starting points, it was possible to identify a global minimum. However, running the optimisation multiple times significantly increased the computational time required to determine the parameters. When applying this technique to other constitutive models, appropriate care is needed to ensure that the global minimum is identified.

In addition, the determination of the sensitivity-based virtual fields requires the user to select an appropriate virtual mesh size and the two reconstruction parameters: the perturbation, $\delta X_i$ and the scaling parameter, $\alpha ^{\left( i\right) }$. For both linear and Voce models, increasing the virtual mesh size only marginally improves the quality of the identification (Tables 4 and 7). The factor limiting the maximum size of the virtual mesh is the available computer memory which is needed to calculate the pseudo-inverse of the modified global strain-displacement matrix, $\overline{\mathbf{B}}$, in Eq. 7. On a computer with 4 GB of RAM, the maximum virtual mesh size that could be used was $20 \times 20$ for a system that includes 17,000 measurement points. For the Voce model, the choice of the perturbation parameter did not have any significant influence on the mean identified values but had a minor influence on the random error (Fig. 17a). The value of the scaling parameter did influence the mean value particularly for the parameters with limited sensitivity (i.e.  *b*, $R_{\mathrm {inf}}$) as seen in Fig. 17b. To implement these sensitivity-based virtual fields, it will be necessary to perform a sensitivity study using simulated data to identify an optimum set of parameters prior to implementing it on experimental data. It is expected however that in the future, when more experience has been gained on different models and test geometries, guidelines can be produced as to the choice of these parameters to avoid searching for appropriate values.

The primary advantage of the virtual fields method over finite element model updating is its computational efficiency. Using a standard PC with an Intel Core i5 processor (3.20 GHz) and 4 GB of RAM memory, the complete identification procedure for the Voce hardening model takes approximately 25, 30, and 35 min using uniform, stiffness-based, and sensitivity-based virtual fields, respectively. Sensitivity-based virtual fields require approximately 20$\%$ more computational time per iteration when compared to the uniform virtual fields. The total time to perform the optimization is heavily dependent on the performance of the radial-return algorithm used to perform the stress reconstruction, keeping in mind that the number of reconstructions increases linearly with the number of model parameters. The times reported here are for a radial-return algorithm in MatLab; however the time spent performing stress reconstructions could be decreased by translating this subroutine into a compiled language. A demo code presenting implementation and general flow of the identification procedure is available in Online Resource 7. It supports all three types of virtual fields used in this work.

Another route to increase computational efficiency is to only selectively update the sensitivity-based virtual fields. For the first several iterations, the virtual fields generated from the initial guess would be used; then, every $n\mathrm {th}$ iteration, the sensitivity-based virtual fields would be updated. If the virtual fields are not updated, they can be carried from the previous iteration directly reducing the number of stress reconstructions that are needed. While not critical for the models tested here which included a maximum of 4 parameters, selective updating will likely to be critical to keep the run time down for models with a large number of parameters. This will be investigated in future studies.

In the future, this method will be tested with more complex non-linear constitutive models. Currently, the sensitivity-based virtual field concept is being extended to large deformation and anisotropic plasticity (Hill48, Yld2000-2D). This will be applied to tests such as presented in [14, 25]. Future work also includes extending the method to dynamic loading [21] where the virtual work due to inertia will be accounted for in Eq. 1. While not addressed in this manuscript, the virtual fields method has already been validated for large strain hyperelasticity [23] and sensitivity-based virtual fields could be used to identify hyperelastic material parameters. It should be noted that for models that do not include history-dependence, it may be appropriate to examine again which of the stress-sensitivity or incremental stress sensitivity performs better for the inverse material parameter identification.

## Data report

No data are provided with this article as only simulations were used which can easily be reproduced from the information in the article.

### Electronic supplementary material

Below is the link to the electronic supplementary material.

A video showing variation of $\bar{\varepsilon}^p$, $\delta\sigma_{22}^{(\sigma_0)}$, and $\delta\tilde{\sigma}_{22}^{(\sigma_0)}$ with time steps for the linear hardening model. Supplementary material 1 (avi 8094 KB)

A video showing variation of $\bar{\varepsilon}^p$, $\delta\sigma_{22}^{(H)}$, and $\delta\tilde{\sigma}_{22}^{(H)}$ with time steps for the linear hardening model. Supplementary material 2 (avi 8863 KB)

A video showing variation of $\bar{\varepsilon}^p$, $\delta\sigma_{22}^{(\sigma_0)}$, and $\delta\tilde{\sigma}_{22}^{(\sigma_0)}$ with time steps for the Voce hardening. Supplementary material 3 (avi 8201 KB)

A video showing variation of $\bar{\varepsilon}^p$, $\delta\sigma_{22}^{(R_0)}$, and $\delta\tilde{\sigma}_{22}^{(R_0)}$ with time steps for the Voce hardening. Supplementary material 4 (avi 8954 KB)

A video showing variation of $\bar{\varepsilon}^p$, $\delta\sigma_{22}^{(R_{inf})}$, and $\delta\tilde{\sigma}_{22}^{(R_{inf})}$ with time steps for the Voce hardening. Supplementary material 5 (avi 8402 KB)

A video showing variation of $\bar{\varepsilon}^p$, $\delta\sigma_{22}^{(b)}$, and $\delta\tilde{\sigma}_{22}^{(b)}$ with time steps for the Voce Hardening. Supplementary material 6 (avi 8019 KB)

Demo code in Matlab presenting implementation of the proposedvirtual fields. Supplementary material 7 (zip 74891 KB)

## Appendix 1

### Virtual mesh: piecewise virtual fields

The use of a virtual mesh, allows the virtual displacement, $\varvec{u}^*$, to be defined in a piecewise manner over the surface of the body. The primary advantage of defining the virtual displacement in a piecewise manner is flexibility; this is particularly useful when defining the sensitivity and stiffness-based virtual fields. The formulation of the piecewise virtual fields is briefly described below; for additional details on implementing piecewise virtual fields see Section 3.6 of [20].

In this work, a virtual mesh consists of isoparametric linear quadrilateral virtual elements defined by four nodes. Each node has two degrees of freedom, the two in-plane virtual displacements. Within an element, virtual displacements are calculated as:

13 $$\begin{aligned} \varvec{u}^{*}(\xi ,\eta ) = \sum _{i=1}^{4} \varvec{N}^{(i)}(\xi ,\eta ) \varvec{u}^{*(i)} \end{aligned}$$

where $\xi$ and $\eta$ are the local (natural) coordinates of each element, $\varvec{N}^{(i)}$ is a shape function of node *i* at point $(\xi , \eta )$, and $\varvec{u}^{*(i)}$ is a nodal value of displacement at node *i*. Strain is calculated as a derivative of displacement with respect to the global coordinate system:

14 $$\begin{aligned} \varvec{\varepsilon }^{*}(x,y)= & {} \sum _{i=1}^{4} \varvec{B}^{(i)}(x,y)\varvec{u}^{*(i)} \nonumber \\= & {} \sum _{i=1}^{4} \varvec{J}^{-1}\varvec{B}^{(i)}(\xi ,\eta )\varvec{u}^{*(i)}, \end{aligned}$$

where *x* and *y* are the global coordinates, ${J_{ij}}=\frac{\partial x_{i}}{\partial \xi _{j}}$ is the Jacobian matrix of local-to-global coordinate transformation, and $\varvec{B}^{(i)}$ is a strain transformation matrix for node *i*. The shape functions then define the virtual displacement over the element and the virtual strains. Boundary conditions are also easily enforced directly at the constrained nodes.

## Appendix 2

### Derivation of stiffness-based virtual fields for quadrilateral elements

Stiffness-based virtual fields were derived by estimating the effect that noise has on the data and developing a special class of virtual fields that minimise the influence of the noise [19]. The stiffness-based virtual fields can be obtained by inverting the following system:

15 $$\begin{aligned} \begin{bmatrix} \varvec{H}&\varvec{\varGamma }^{T} \\ \varvec{\varGamma }&\varvec{0} \end{bmatrix} \begin{bmatrix} \varvec{u}^{*} \\ \varvec{\lambda } \end{bmatrix} = \begin{bmatrix} \varvec{0} \\ \varvec{u}_{BC} \end{bmatrix} \end{aligned}$$

where $\varvec{H}$ is the matrix derived from the tangent stiffness matrix, $\varvec{\lambda }$ is a vector containing Lagrangian multipliers, $\varvec{u}_{BC}$ is a vector containing values of the prescribed displacements and $\varvec{\varGamma }$ is a matrix containing prescribed virtual boundary conditions. For the specific case presented in Sect. 4.1, the vertical displacements at the top boundary are equal to *L*, the length of specimen, and the displacement at the bottom boundary is equal to 0. Matrix $\varvec{H}$ is obtained from:

16 $$\begin{aligned} \varvec{H} = \varvec{K}^{*} \varvec{K}^{*} \end{aligned}$$

where $\varvec{K}^{*}$ is the modified global stiffness matrix, as defined in Eq. 17. It depends on *n*, the number of virtual elements, *t*, the thickness of specimen, $\sigma ^{eq}$, the effective von Mises stress, $\varvec{B}$, the strain-displacement matrix, and $\varvec{D}^{ep}$, the tangential elasto-plastic stiffness matrix.

17 $$\begin{aligned} \varvec{K}^{*} = \sum _{elem=1}^{n} \int _{A}\; t\, \sigma ^{eq}\, \left( \varvec{B}^{T} \varvec{D}^{ep} \varvec{B}\right) \, dA \end{aligned}$$

There are a few differences between the original formulation given in Pierron et al. [19] and the implementation used in this manuscript. Originally, the experimental data were interpolated onto a grid consisting of triangular elements and, as a result, the virtual elements were constant strain triangular elements. To be consistent with the quadrilateral mesh used for the sensitivity-based virtual fields, the method for the stiffness-based virtual fields was reformulated for a quadrilateral mesh. For a linear quadrilateral element, the integration of the stiffness matrix is not a trivial task unless the strains are known at the Gauss (integration) points. However, this is not generally the case. To avoid interpolating the strains at the Gauss points and performing additional stress reconstructions at the Gauss points, another approach was taken. The tangential elasto-plastic stiffness matrix, $\varvec{D}^{ep}$, was computed as an average value across each element taking into account all measurements points within an element. For the radial-return algorithm used in this work, the consistent elasto-plastic tangential stiffness matrix for von Mises plasticity with isotropic hardening is [16]:

18 $$\begin{aligned} \varvec{D}^{ep}&= \varvec{E} - \alpha (\varvec{E}\varvec{P}\varvec{\sigma }_{n+1}) \otimes (\varvec{E}\varvec{P}\varvec{\sigma }_{n+1}) \nonumber \\ \varvec{E}&= \left[ \varvec{C}+\varDelta \gamma \varvec{P} \right] ^{-1} \nonumber \\ \alpha&= \frac{1}{\varvec{\sigma }_{n+1}^{T} \varvec{P} \varvec{E} \varvec{P} \varvec{\sigma }_{n+1} - \frac{2\sigma ^{eq} d\sigma _{y}/d\bar{\varepsilon }^{p}}{3-2d\sigma _{y}/d\bar{\varepsilon }^{p}\varDelta \gamma }} \end{aligned}$$

where $\varvec{C}$ is the plane stress compliance matrix, $\varvec{P}$ is a matrix mapping a stress vector to the equivalent stress (Eq. 19). $\varDelta \gamma$ is the plastic multiplier obtained in a implicit Newton-Raphson radial return scheme, $\sigma ^{eq}$ is the equivalent stress, $\sigma _{y}$ is hardening law and $\otimes$ is a dyadic product of two vectors.

19 $$\begin{aligned} \left( \sigma ^{eq}\right) ^{2} = \frac{3}{2} \varvec{\sigma }^{T} \varvec{P} \varvec{\sigma }, \quad \varvec{P} = \frac{1}{3} \left[ \begin{matrix} 2 &{} -1 &{} 0 \\ -1 &{} 2 &{} 0 \\ 0 &{} 0 &{} 6 \end{matrix} \right] \end{aligned}$$

This averaged tangential elasto-plastic stiffness matrix was assumed to be valid at the centroid of the element and the element stiffness matrix was integrated using the standard reduced integration scheme for a 4-noded quadrilateral. The effective von Mises stress was also calculated by taking the average value across the element. Note that this simplification is not physical, but since the virtual fields are just mathematical test functions, it is a reasonable simplification. This approach of taking the average value across each virtual element limits the coarseness of the stiffness-based virtual fields. If the virtual elements become too large the assumption of using the average value at the centroid will lead to inappropriate virtual fields.
